# Advanced fabrication approach for innovative triple base propellants with enhanced continuous fracture resistance†

**DOI:** 10.1039/d3ra04828d

**Published:** 2023-12-06

**Authors:** Yao Zhu, You Fu, Bin Xu, Zhitao Liu, Feiyun Chen, Xiaoan Wei, Xin Liao

**Affiliations:** a School of Chemistry and Chemical Engineering, Nanjing University of Science and Technology Nanjing Jiangsu 210094 China howtosci@163.com liaoxin331@163.com lzt03304645@163.com; b Key Laboratory of Special Energy Materials, Ministry of Education Nanjing Jiangsu 210094 China

## Abstract

This paper initially contrasts the solvent-based and solventless molding processes, subsequently optimizing a sustainable and efficient solventless molding route for both STP and SLTP. Key physicochemical parameters such as extrusion rate, residual volatile solvents, moisture content, and apparent density of both propellant types are meticulously compared. Furthermore, the orientation of crystal particles and the structure of the matrix–bound interface are analyzed. Comprehensive examination of triaxial progressive failure phenomena—including static thermal mechanical responses, quasi-static structural deformation, and dynamic structural damage—is conducted, leading to the formulation of a damage mechanism and model. Subsequently, a structural mechanics model for nitroguanidine micrometer rod-reinforced triple base propellants is established, quantitatively evaluating the influence of nitroguanidine crystal arrangement angles on the structural strength of both propellant types. This study furnishes a theoretical foundation for specialized internal structural and mechanical behaviors through theoretical computations.

## Abstract 2

This research optimizes the production of triple base propellants, comparing solvent-based and solvent-free processes. Solventless propellant exhibits superior environmental adaptability, dynamic impact endurance, and fragmentation resistance.

## Graphical abstract

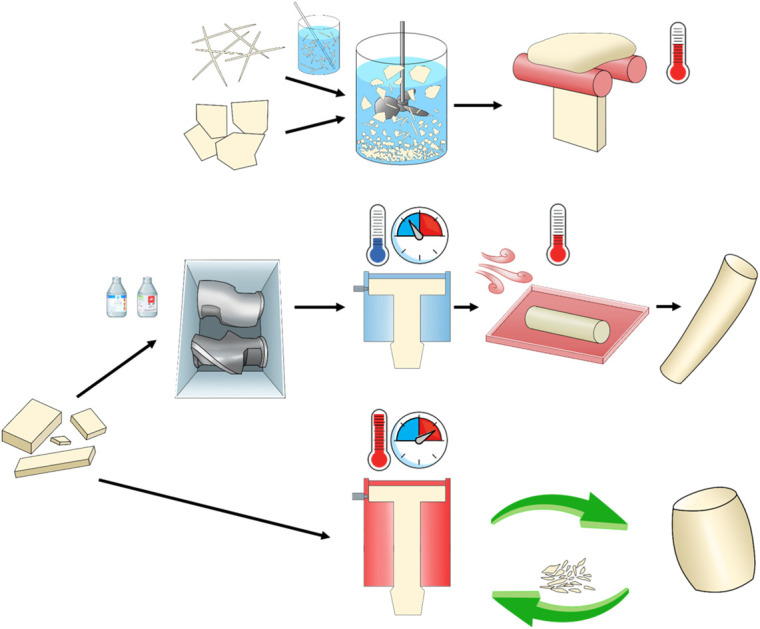

## 1 Introduction

A triple base propellant, exemplified by M30, consists of a high-energy solid filler, plasticizer, and an energetic composite binder skeleton. It employs the adhesion of energetic material composite systems to forge the solid structure. This structure is vulnerable to simultaneous local and overall internal stresses stemming from various adverse factors, including the shrinkage effect of volatile additives, temperature-induced embrittlement, solid filler debonding, and flawed molding processes, which frequently subject the organizational structure to stress loading.^1–3^

The formulation and the preparation process are both critical to the performance of the propellant. The solvent type method, widely used for triple base propellants, is recognized for its dependable processes.^4^ Nevertheless, in the context of developing new “green energetic composite materials” and the imperative for sustainable practices within the defense science and technology industry, the solvent molding process for propellants must be urgently optimized. This is primarily due to the lengthy processing cycles, high energy consumption, and suboptimal productivity currently observed.^5–8^ During the solvent-based process, a volatile co-solvent is typically added during gelatinization and subsequently removed through drying, a process that is energy-intensive and not always effective in completely eliminating the co-solvent. Concurrently, significant quantities of co-solvent are evaporated during the solvent removal phase, leading to substantial raw material wastage and environmental contamination, as well as the loss of valuable processing time. Moreover, this method can result in product quality issues such as uneven density, dimensional inconsistency, and internal cavities.^9^ On the other hand, the solventless process produces a dimensionally more stable product since gelatinization occurs without solvent involvement, making it preferable for manufacturing larger grains.^10–12^

The solventless molding process boasts several benefits, including reduced operation time, decreased energy consumption, lower emissions, and enhanced consistency in the quality of complex-shaped, various-sized, high-quality propellants. This method represents a superior approach to propellant manufacturing, addressing issues such as shape shrinkage, uneven density distribution, and dimensional inconsistency that are associated with the co-solvent in the solvent-based molding process.^13–16^ Moreover, the high-pressure application during the solventless molding enhances the energy density and dimensional precision of the product, optimizes the filler particle orientation distribution, and strengthens the internal structure's bond. Furthermore, this process is a component of green manufacturing technologies for energetic composite materials, embodying a modern manufacturing approach that thoroughly accounts for environmental impacts, resource consumption, and production efficiency, while also prioritizing safety and security.^17–19^

The solventless molding process eschews the use of volatile co-solvents, instead leveraging the dissolving and plasticizing capabilities of nitrate on nitrocellulose. This method employs energetic composites plasticizer as the binder and utilizes integrated high-temperature and high-pressure extrusion molding. Owing to the absence of volatile co-solvents, this process negates the need for co-solvent repulsion and additional hardening procedures, thus enabling the reduction of numerous process cycles, curtailing energy losses, and diminishing hazardous waste emissions, among other processing benefits. Furthermore, the application of high pressure in the solventless molding process serves to augment the energy density and dimensional precision of the product, as well as to improve the orientation of filler particles and the bonding strength within the internal structure. The solventless type molding process route presents several advantages, including reduced material usage, a shorter path, lower emissions, higher efficiency, stability, and reliability. It is considered a more efficient and modern manufacturing process route for propellants.^20–22^

In this study, we conducted a comparative analysis between solvent-based and solventless molding processes, subsequently refining an eco-friendlier and more efficient solventless molding process for fabricating STP and SLTP. We assessed macroscopic physicochemical properties, including the extrusion swelling rate, residual volatile solvents, moisture content, and apparent density of both propellant types. Additionally, we examined the microstructural morphology through the orientation distribution of crystal particles and the structure of the matrix–bound interface. Addressing the observed radial mechanical weaknesses in the novel triple base propellant, we explored its progressive failure behaviors, such as static and dynamic thermo mechanical properties (TMA and DMA) and crush resistance under extreme conditions. Finally, we developed a structural mechanics model for nitroguanidine micrometer rod-reinforced triple base propellants, quantifying the impact of the solid-filled nitroguanidine crystals' arrangement angles on the structural strength of both types of propellants. This work also lays a theoretical groundwork for understanding the special internal organization and mechanical behaviors *via* theoretical computations.

## 2 Experimental sections

### 2.1 Materials and processing

The composites consist of nitrocellulose (NC), which absorbs nitroglycerin (NG) and diethylene glycol dinitrate (DEGDN), exhibiting a pasty state amenable to rolling processes. The paste composition includes 60.0 wt% NC at a nitration level of 12.5%, 28.0 wt% NG, 9.5 wt% DEGDN, 2.0 wt% dimethyl phenyl urea (C_2_), and 0.5 wt% TiO_2_, along with Nitroguanidine (NQ) of 99% purity. Analytical reagent-grade acetone, ethanol in a 1 : 0.95 v/v ratio, and the surfactant emulsifier OP-10 were also employed. All chemicals were utilized as received, without further purification or modification.

### 2.2 Composite manufacture

Initially, a pre-dispersion process of pure needle-shaped NQ was conducted to ensure its uniform distribution within the propellant matrix. An aqueous suspension containing 20.0 wt% pure needle-shaped NQ in 5000 mL of deionized water was prepared, stirred magnetically for 30 minutes, and then subjected to ultrasonic treatment at 25 °C for another 30 minutes. To enhance the dispersion of needle-shaped NQ, 5 mL of emulsifier OP-10 was dissolved in 10 mL of ethanol, functioning as both surfactant and dispersant. Subsequently, 80.0 wt% of a NC/NG/DEGDN composite paste was incorporated into this aqueous suspension. The mixture was exposed to ultrasonic dispersion at 50 kHz and mechanical stirring at 2800 rpm for 5 hours. This process ensured the NC/NG/DEGDN paste was homogeneously dispersed amidst the needle-shaped NQ particles, effectively disaggregating initial clumps and promoting the absorption of NQ by the composite paste. After complete natural sedimentation of the suspension, the clear supernatant was decanted. The resultant paste from the sediment was then calendared into sheets at 80 °C and subsequently cured in an oven at 50 °C for seven days to eliminate any residual solvent and moisture. The resulting triple-base propellant material incorporated approximately 20.0% needle-shaped NQ within the NC/NG/DEGDN matrix.

**Fig. 1:**
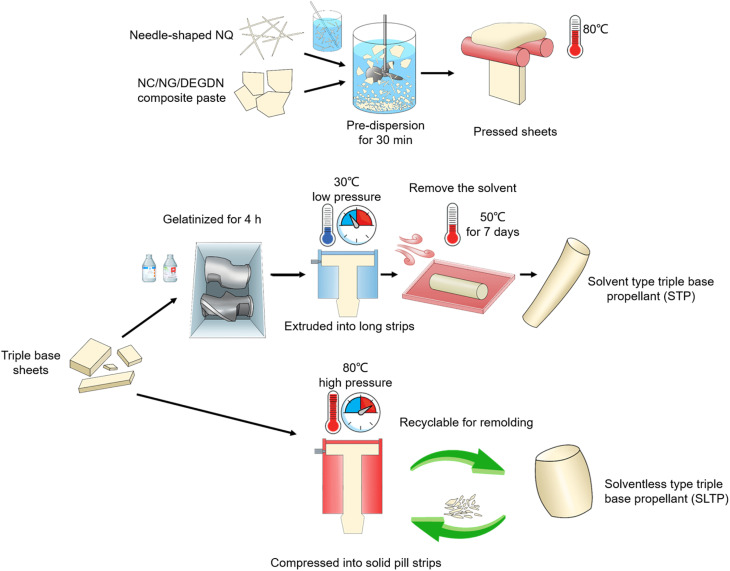
Schematic diagram of process fabrication of STP and SLTP composite samples.

Subsequently, half of the prepared triple-base propellant material was blended with a rich solvent to form the STP. This mixture was agitated in a blade incorporator equipped with a cooling water bath and gelatinized over a period of 4 hours, followed by extrusion into elongated strips using an extrusion molding technique. The STP samples, shaped into pillars, were then placed in a drying oven at 45 °C for 8.3 days to ensure complete solvent evaporation. Conversely, the remaining half of the triple-base propellant material was subjected to a high-temperature and high-pressure process for 0.2 hours to induce softening and plasticization without utilizing any solvents, resulting in the solventless type triple-base propellant (SLTP). This softened mass was then formed into solid pill strips using the same extrusion molding approach. The SLTP pillar samples were preserved in sealed bags at a consistent room temperature of 25 °C. Fig. 1 illustrates the schematic process flow for fabricating both the STP and SLTP strands.

Experimental parameters and the compositions of STP and SLTP with a different gelating production method were shown in Table 1.

**Table 1:** Sample parameters and the compositions of STP and SLTP with a different gelating production method

Parameter	STP			SLTP		
	1#	2#	3#	4#	5#	6#
Gelation time (h)	4	4	4	0.2	0.2	0.2
Molding temperature (°C)	35	35	35	70	80	90
Extrusion pressure (MPa)	6.62	5.53	4.85	64.83	51.37	38.68
Curing time (*d*)	>8.33	>8.33	>8.33	0	0	0

**Fig. 2:**
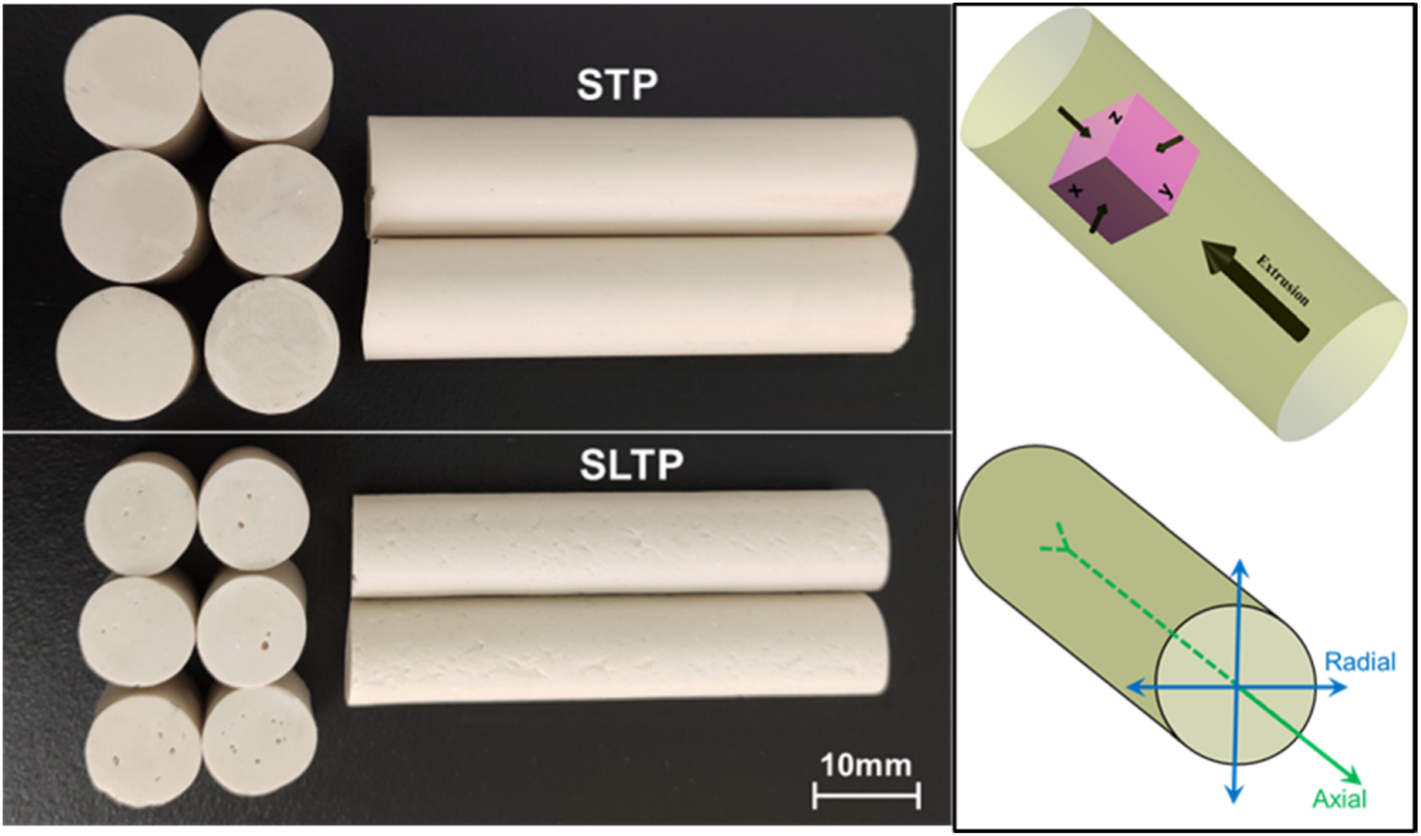
Photos of triple-base propellants with the mutual relationship between extrusion molding direction and tri-axial test loading direction.

Thirdly, the two aforementioned propellant samples were cut into cylindrical, cubic, and dumbbell-shaped long sticks for mechanical property testing, including static thermo mechanical analysis, dynamic thermo mechanical analysis, and collision crushing strength assessments. Fig. 2 is the photographs of the triple-base propellants produced *via* different plasticization methods. Observing the transverse sections in Fig. 2, a slight inconsistency in the diameter of the STP and SLTP propellants can be discerned. The fabrication of these propellants was conducted in compliance with the protocols delineated in the General Specification for Propellant GJB 1529A-2001.

### 2.3 Characterization

In this research, the specimens underwent comprehensive characterization and testing using methods such as scanning electron microscopy (SEM), the density bottle technique, static mechanical thermal analysis (TMA), dynamic mechanical thermal analysis (DMA), and a collision crushing strength test system. Detailed descriptions of the characterizations and testing methodologies are provided in the ESI† section.

## 3 Results and discussion

### 3.1 Macroscopic physicochemical parameters

Physicochemical parameters of the STP and SLTP samples are presented in Table 2.

**Table 2:** Physico-chemical parameters of STP and SLTP

Physico-chemical parameters	STP			SLTP		
	1#	2#	3#	4#	5#	6#
Ratio of expansion (%)	1.92	1.64	1.25	2.73	2.54	2.27
VOCs residues (wt%)	0.89	0.96	1.21	0.03	0.05	0.03
Moisture residue (wt%)	1.23	1.16	1.27	0.73	0.61	0.49
Apparent density (g cm^−3^)	1.59	1.55	1.53	1.68	1.65	1.62

The extrusion expansion ratios for SLTP samples surpassed those of the STP samples. Additionally, the expansion ratios for both SLTP and STP samples increased proportionally with the rise in extrusion pressure. Specifically, samples 4#, 5#, and 6# exhibited expansion rates of 2.73%, 2.54%, and 2.27% respectively, while samples 1#, 2#, and 3# had rates of 1.92%, 1.64%, and 1.25%. Evidently, the extrusion molding process is propelled by the differential pressure, which is stabilized and then released at the mold's outlet, causing the material to expand upon exit. The higher extrusion expansion rate for SLTP samples can be ascribed to their differential pressure being approximately tenfold that of STP samples. In contrast, STP samples, laden with volatile industrial additives, undergo significant shrinkage and hardening as these additives are expelled, resulting in a lower expansion rate. Conversely, the scarcity of volatile additives in SLTP samples means shrinkage is largely absent, leading to a higher rate of expansion post-extrusion.

The volatile organic compounds (VOC) residues in SLTP samples were found to be less than those in STP samples. Notably, the residual concentration of VOCs in STP samples escalated with an increase in the solvent mass fraction during the processing parameters. Specifically, the residual VOC concentrations in samples 1#, 2#, and 3# were 0.89 wt%, 0.96 wt%, and 1.21 wt%, respectively. In contrast, samples 4#, 5#, and 6# had significantly lower residual VOC concentrations at 0.03 wt%, 0.05 wt%, and 0.03 wt%, respectively. These values are within the range of acceptable measurement error, suggesting that the SLTP samples are virtually devoid of VOC residues. The diffusion of VOCs is characteristic of a diffuser–polymer system, with higher concentrations initially present within the STP sample's cylinder. The VOCs tend to migrate outward from the core to the surface of the STP sample, propelled by the interplay of concentration gradients, temperature, and time. Consequently, as VOC concentrations diminish, the expulsion efficiency decreases, complicating the attainment of complete VOC removal.

The moisture residual concentrations in SLTP samples were consistently found to be lower than those in STP samples. Furthermore, the detected moisture residuals in STP samples exceeded 1%, whereas those in SLTP samples remained below 0.8%, with a noticeable decrease in moisture residual concentration as the molding temperature increased. Specifically, samples 1#, 2#, and 3# had moisture residuals of 1.23 wt%, 1.16 wt%, and 1.27 wt%, respectively. In contrast, samples 4#, 5#, and 6# exhibited significantly lower moisture residuals at 0.73 wt%, 0.61 wt%, and 0.49 wt%, respectively. The STP process, characterized by solvent plasticization molding, tends to operate at lower temperatures, resulting in higher extrusion efficiency but also higher moisture retention. The SLTP process, on the other hand, employs high-temperature plasticizing molding, leading to elevated molding temperatures, reduced extrusion efficiency, but also diminished moisture residue.

The apparent densities of the SLTP samples were consistently higher than those of the STP samples. Notably, the surface of the SLTP sample presented as glossy and robust, in contrast to the STP sample's wrinkled surface, which exhibited a number of pores. Additionally, the apparent densities of both SLTP and STP samples displayed a tendency to increment alongside the increase in molding pressure. Specifically, the apparent densities for samples 4#, 5#, and 6# were recorded at 1.68%, 1.65%, and 1.62% respectively, while those for samples 1#, 2#, and 3# were 1.59%, 1.55%, and 1.53%. As anticipated, the high-pressure extrusion process effectively expunges gas cavities, resulting in a denser material with components that are more closely interlinked. The STP samples, due to the expulsion of industrial additives, experienced considerable shrinkage and hardening of the extruded material, leading to a peeling effect at the bonding interfaces between the needle-shaped NQ crystals and the matrix. This process creates a micro-interfacial layer and diminishes the apparent density of STP samples. In stark contrast, the SLTP samples undergo the extrusion process with negligible shrinkage, ensuring that the bonding interfaces between the needle-shaped NQ crystals and the matrix remain firmly connected, thereby contributing to the higher apparent density of the SLTP samples.

### 3.2 Microstructure morphology

#### 3.2.1 Orientation distribution

The industrial-grade raw nitroguanidine presents as a white, smooth, low-density powder on a macroscopic scale. The SEM image provides an insightful perspective on the microstructure of these industrial-grade nitroguanidine crystals (Fig. 4). They exhibit a needle-shaped, longitudinal morphology, with lengths ranging from approximately 20–80 μm and diameters spanning about 2–8 μm.

**Fig. 3:**
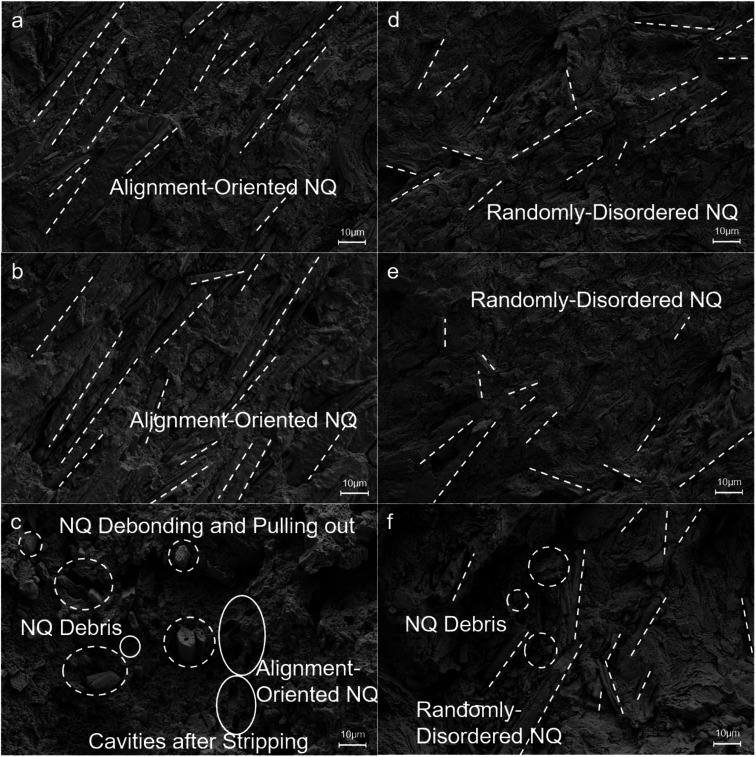
Completely different microscopic morphologies and spatial structure on the fracture surface morphology of STP samples and SLTP samples at a magnification of 3k×. ((a–c) correspond to the *X*, *Y*, *Z* acquisition planes of the STP samples; (d–f) correspond to the *X*, *Y*, *Z* acquisition planes of the SLTP samples, respectively).

Fig. 3 provides a comparative analysis of the impact of long needle-shaped NQ on the orientation distribution within various propellant samples. The study involved measuring the diameters of the long needle-shaped NQ and their orientation to establish an orientation distribution for STP and SLTP samples. The diameters of the NQ within the STP and SLTP samples were consistent with that of the raw NQ; however, there was a notable difference in length, as depicted in Fig. 3. Within the spatially supported structure of the SLTP samples, the long needle-shaped NQ measured 10–20 μm in length. In contrast, the STP samples, featuring a planar laminar structure, contained long needle-shaped NQ particles of 30–50 μm in length, with a presence of uniformly dispersed shorter NQ particles also observed. Additionally, remnants of NQ debris were detected on the surfaces of the fractured composite matrix. This suggests that the STP samples experienced shear failure around the pulled-out NQ particles, which implies a less effective role in the toughening effect.

**Fig. 4:**
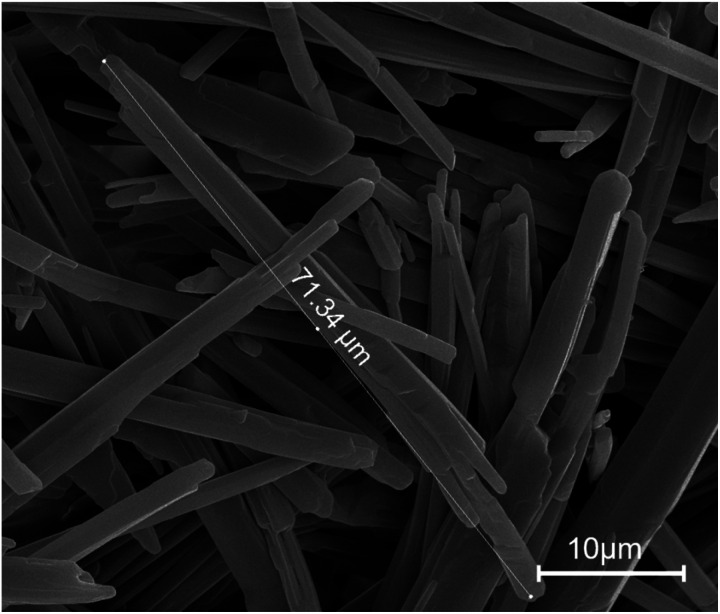
SEM images of pure needle-shaped nitroguanidine.

Fig. 3 reveals that the SEM patterns of the longitudinal and transverse cross-sections of the propellant cylinders exhibit significant disparities relative to the axis of the cylinder. This can be explained by the extrusion process utilized for both the STP and SLTP propellant grains. During the process, the grains are extruded through an annular capillary die, resulting in a non-uniform distribution of shear rates and velocities characteristic of Poiseuille flow, with higher shear rates occurring near the die walls. For STP samples, the needle-shaped NQ crystals are compelled to align with the flow direction (axially) under compression pressure, leading to clusters of NQ crystals that exhibit a high degree of uniform orientation. In contrast, the needle-shaped NQ crystals within the SLTP samples are unable to achieve such organized orientation due to the substantial surface friction and viscosity-related flow constraints during extrusion. Concurrently, NQ crystals that exhibit minor radial fissures are broken radially by intense shear stress, fragmenting into rod-shaped particles that are resistant to further breakage.

The SLTP samples exhibit fewer instances of pull-out and a more robust interface between the NQ and the matrix. In contrast, the STP samples present with notable deep holes, approximately 5 μm in diameter, indicative of the pull-out of long needle-shaped NQ. Furthermore, the STP samples display broken, long needle-shaped NQ with extensive pull-outs aligned in the direction of the load and protruding from the fracture surface, perpendicular to the notch. The predominantly clean surfaces of the long, needle-shaped NQ within the STP samples suggest a weak bond at the interfaces. While similar breakage of long needle-shaped NQ is evident in the SLTP samples, these are typically not aligned with the loading direction. Most are off-axis, which mitigates the likelihood of NQ breakage and resultant pull-out, leading to a significantly lower incidence of NQ pull-outs due to enhanced adhesion to the matrix. It is likely that the separation of these long needle-shaped NQ from the matrix in SLTP samples is due to transverse-shear loading. This phenomenon is attributed to the increased level of NQ-matrix adhesion present in SLTP samples, which facilitates greater stress distribution and results in reduced debonding, NQ pull-outs, and deformation.^23^

The triple-base propellant samples manufactured through the two distinct molding processes exhibit divergent microscopic morphologies and spatial arrangements. As depicted in Fig. 3, within the left and right columns, there is a stark contrast between the morphologies of STP and SLTP samples. The needle-shaped NQ crystals in STP samples are precisely aligned, with their longitudinal axes connected end-to-end, paralleling one another. In contrast, the needle-shaped NQ crystals in SLTP samples display a random orientation, arranged haphazardly in both horizontal and vertical planes, providing mutual support. This interspersion of NQ rods is enveloped by the NC/NG/DEGDN matrix, which results in a three-dimensional spatial structure that includes numerous stable “triangular” and “fence-like” configurations, conducive to effective force transfer and exemplary mechanical properties. This intricate spatial structure aids in force dispersion. On the other hand, the STP specimens are characterized by a significant planar laminar structure, which is less effective in dispersing and cushioning stresses. It is evident that the crystal morphology and spatial configuration of NQ are critical in determining the mechanical integrity of the propellants.^24,25^

### 3.3 Thermo mechanical response

#### 3.3.1 Static thermo mechanical properties

Before commencing the thermal stability investigation, the thermogravimetric analyzer underwent calibration for both temperature and weight precision. This was achieved by employing the melt enthalpy standard indium (GBWE130128) with a known melting point of 156.52 °C, F1 grade stainless steel standard weights for accuracy in mass measurement, and standard nickel (GBW13240) to calibrate the Curie temperature at 358.6 °C.

The pure NQ tablet sample demonstrated a negative coefficient of thermal expansion, aligning with findings from previous research. The molecular arrangement within NQ could explain the observed negative thermal expansion in these pure NQ tablet samples. In a NQ molecule, the conjugate effect creates a connection involving a single carbon atom bonded to three nitrogen atoms. Due to the presence of potent hydrogen bond donors (nitro groups) and acceptors (amino groups), robust networks of hydrogen bonds are likely to form, facilitating the two-dimensional packing of NQ molecules through intermolecular hydrogen bonding. With rising temperatures, the hydrogen bonds tend to relax, diminishing steric hindrance between molecular layers, thereby contracting the perpendicular interlayer spacing. The thermal expansion behavior of NQ crystals is distinctively anisotropic, which could be attributed to the anisotropic nature of the intermolecular forces.^26,27^

During routine assessments of the linear thermal expansion coefficients of STP and SLTP samples, a notable difference in expansion between the two types of propellants was observed. The STP samples exhibited a higher coefficient of expansion in the *X* and *Y* planes when compared to the *Z* plane, with a significant margin of approximately 90.00 × 10^−6^ K^−1^. This outcome is particularly unexpected given that the STP samples displayed more pronounced expansion in the *Z* acquisition plane, deviating from the norm. Furthermore, the thermal expansion profiles of the SLTP samples contrast with those of the STP samples, indicating that the thermal behavior of the SLTP material encapsulates two distinct phases.

It is apparent that in the initial phase, which spans approximately from −50 to 48.83 °C, the coefficient of linear expansion is greater. In the subsequent phase, beginning at roughly 48.83 °C, the coefficient of linear expansion diminishes, with an inflection point occurring around this temperature. Consequently, this inflection point where the coefficient transitions from high to low is indicative of the glass transition temperature of the SLTP sample. This transition also signifies the softening of the SLTP sample at the inflection temperature. Notably, the glass transition temperature for the SLTP samples remains stable irrespective of the plane of detection. The coefficients of linear expansion across the *X*, *Y*, and *Z* acquisition planes exhibit a high degree of overlap and remarkable uniformity throughout the entire temperature range.

**Fig. 5:**
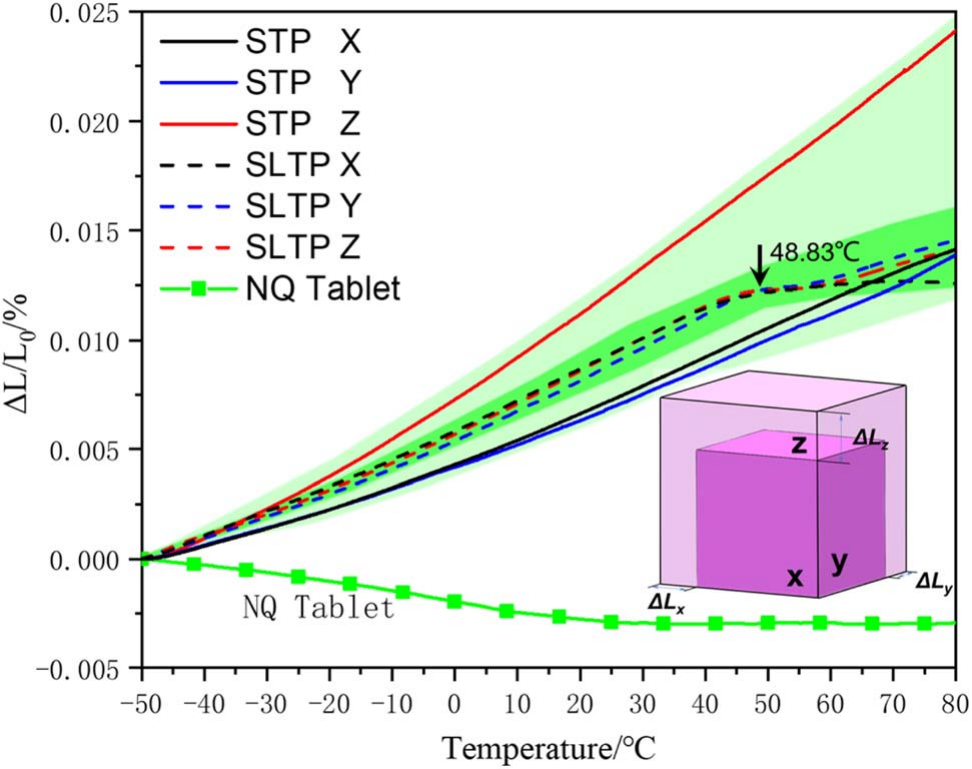
Thermal expansion curves of the STP and SLTP samples in three acquisition planes (*X*, *Y*, *Z*) with mutual perpendicularity and their corresponding glass transition temperature (*T*_g_).

During the variable temperature experiments, a progressive reduction in the thickness of the pure NQ tablet was observed as the temperature was raised from −50 °C to 80 °C. Conversely, for the *X* and *Y* test planes, the thickness increased slowly, while for the *Z* test plane, there was a rapid increase in thickness, as depicted in Fig. 5. This phenomenon suggests that the highly uniform orientation of the NQ crystals within the STP samples contributes to marked anisotropy in thermal expansion across the three mutually perpendicular acquisition planes. As shown in Table 3, within the −50 °C to 80 °C temperature range, the average thermal expansion coefficient of STP samples along the *Z* plane was substantially higher, ranging approximately from 145–220 × 10^−6^ K^−1^. In contrast, the average thermal expansion coefficients along the *X* and *Y* planes were considerably lower, estimated between 83–141 × 10^−6^ K^−1^. Interestingly, the average thermal expansion coefficients of the SLTP samples along the *X*, *Y*, and *Z* planes were highly consistent, falling between the aforementioned ranges, at approximately 99–172 × 10^−6^ K^−1^.

**Table 3:** Linear expansion coefficients of STP and SLTP samples in three collection planes (*X*, *Y*, *Z*) with mutual perpendicularity

Specimens	Acquisition plane	Coefficient of linear expansion/10^−6^ K^−1^				
		−50–0 °C	0–20 °C	20–40 °C	40–60 °C	60–80 °C
NQ tablet	—	−39.15	−40.41	−10.54	2.09	−0.07
STP	*X*	85.94	111.36	128.34	132.55	141.26
	*Y*	83.53	106.12	120.33	121.33	133.38
	*Z*	145.52	193.53	209.94	208.01	220.08
SLTP	*X*	99.19	120.91	118.01	43.32	2.69
	*Y*	122.85	156.49	172.96	89.43	100.56
	*Z*	113.79	143.97	147.01	50.50	76.00

In SLTP samples with high viscosity, the orientation of the NQ needles tends towards an isotropic distribution, resulting in isotropic thermal expansion properties. Conversely, in SLTP samples with low viscosity, the NQ needles exhibit uniaxial alignment, leading to a higher proportion of molecules oriented in a specific direction, which in turn causes a pronounced directional thermal expansion in STP samples. The anisotropic nature of the thermal expansion in STP samples underscores the significant influence of the alignment orientation of the NQ on the thermomechanical response of the matrix.

#### 3.3.2 Dynamic thermo mechanical properties

Dynamic mechanical thermal analysis (DMA) is commonly employed to examine the interplay between the microstructure of materials and their dynamic mechanical properties. In the realm of complex energetic polymer composites, DMA is instrumental in yielding insights not only into the viscoelastic characteristics of the composite but also into the dispersion of the filler within the polymer matrix and the resultant microstructure. Solid propellants are categorized as viscoelastic materials, which means that when they are exposed to stress, a portion of the applied force is allocated to elastic deformation, while the remainder is dissipated as thermal energy.^28,29^

**Fig. 6:**
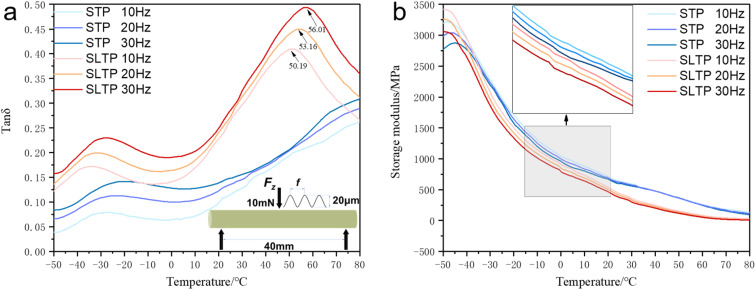
Loss angle tangent curves and energy storage modulus curves of the STP samples and SLTP samples with different driving frequencies in the fully-temperature range.

Dynamic mechanical thermal analysis (DMA) of the STP and SLTP samples was conducted to delve deeper into the interfacial interactions between the NQ and the matrix. The loss angle tangent (tan *θ*) plot depicted in Fig. 6 reveals two distinct mechanical relaxation processes at low and high temperatures for the SLTP sample, in contrast to the STP sample, which exhibits only one relaxation process at low temperatures. For comparative purposes, the loss modulus tan *θ* curves for both STP and SLTP samples at frequencies of 10 Hz, 20 Hz, and 30 Hz are illustrated in Fig. 6.

From the observations in Fig. 6, a consistent pattern emerges regarding the variation of the energy storage modulus with frequency under different temperature conditions for both STP and SLTP samples, with a notable characteristic: the energy storage modulus of the SLTP sample surpasses that of the STP sample within the stable testing temperature range. Across all samples, the energy storage modulus demonstrates a tendency to decline as the temperature rises. Specifically, for both STP and SLTP samples, there is a rapid decrease in energy storage modulus with increasing temperature in the −50 to 0 °C range. As the temperature continues to rise from 0 to 80 °C, the decline in energy storage modulus becomes more gradual. In the lower temperature bracket (−50 to 20 °C), the energy storage modulus decreases with an increase in frequency. However, beyond 20 °C, the energy storage modulus's responsiveness to frequency increments is not as pronounced.

Fig. 6 illustrates comparable trends in the loss factors with frequency for both STP and SLTP samples across varying temperature conditions. In the defined stable test frequency range, the loss factors for both STP and SLTP samples escalate with an increase in frequency. At lower frequencies, there is a reduced number of molecular chains in motion, which correlates with lesser intermolecular interactions. As the frequency is heightened, the macromolecular chains transition out of a static state, with the “thawing” degree of these chains augmenting in tandem with the frequency. Consequently, the increased movement of macromolecules amplifies the internal friction and mechanical loss, leading to an elevation in the loss factor with rising frequency. This increment in loss factor also points to partial microphase separation within the internal structure of STP samples, while simultaneously indicating superior interfacial interactions within SLTP samples compared to their STP counterparts. The robust bond between the NQ and the matrix in SLTP samples is evidenced by the prevention of NQ particle detachment from the matrix surface. When the loading action's frequency is low, the material's internal macromolecular chains stay inert, transmitting force predominantly *via* the movement of smaller chains and segments. However, as the frequency increases, the alternating load action's period becomes shorter than the relaxation time of the molecular chains and segments, rendering their movement unable to keep pace with the stress changes. This results in a macroscopic increase in the energy storage modulus and a concurrent rise in material rigidity. Conversely, at elevated frequencies, the molecular chains are active, and the entangled chains and segments extend. The participation of more molecular chains and the elongation of chain segments heighten the intermolecular friction, thus escalating mechanical loss and leading to a reduction in the energy storage modulus, which ultimately diminishes the material's rigidity.^30^

Furthermore, at a set frequency, the loss factor of SLTP samples exhibits two pronounced loss peaks: one within the −40 to −20 °C range and another within the 50 to 60 °C range, aligning with the findings from TMA analysis. In contrast, STP samples do not present a notable secondary peak across the temperature ranges studied. The loss factor for STP samples displays a single significant peak, occurring within the −30 to 20 °C interval. No new loss peak corresponding to the SLTP sample is identified within the higher temperature spectrum, suggesting that the STP samples lack a distinct glass transition temperature, corroborating the TMA analysis results. With an increase in the test frequency, the peak positions of the loss factors for all samples shift towards higher temperature zones.

The observed peak within the 50–60 °C range is interpreted as the glass transition temperature of the composite system, while the peak within the −40 to 20 °C range is indicative of the embrittlement temperature of the system. This suggests that the embrittlement temperature of SLTP samples is lower, thereby extending their operational temperature range and potentially enhancing their performance across a wider spectrum of environmental conditions.

The loss factor–temperature curve of the SLTP sample distinctly exhibits two substantial decrements as the temperature rises. The first diminution in the loss factor results from the internal components of the SLTP sample transitioning from a crystalline to an amorphous state, which reduces the internal friction among molecular chains and consequently lowers the peak of the loss factor. The subsequent decrease in the loss factor is linked to the glass transition of the amorphous state, where intermolecular friction within the molecular chains weakens, leading to a further decline in the loss peak. In contrast, the STP samples show only one marked decrease in the loss factor, correlating with their internal components' transition from crystalline to amorphous state, absent a pronounced glass transition. This observation corroborates the TMA findings previously discussed.

The glass transition temperature (*T*_g_) is indicative of the transition of the amorphous phase in energetic composites to a glassy state. The *T*_g_ is a pivotal characteristic for energetic composites, as it not only mirrors the dynamic behavior of the composite molecular chains but also influences thermoforming parameters of such materials. When the temperature exceeds *T*_g_, the composite system transitions from a glassy to a viscous flow state, characterized by minimal frictional heat generation, which is optimal for the extrusion molding of energetic composite materials. Additionally, *T*_g_ escalates with an increase in actuation frequency. This is because the vicinity of *T*_g_ marks the culmination of the glass transition region for the SLTP sample, where chain mobility is at its peak and frictional losses are minimal. Beyond *T*_g_, the molecular chains enter a complete viscous flow state, enhancing their mobility and reducing friction loss. A lower loss factor implies reduced hysteresis loss and endogenous heat generation, indicative of superior bonding performance within the composite components.^31^

At low temperatures, the energy of molecular thermal motion is minimal, rendering the thermal motion of the chain segments insufficient to surmount the potential rotational barriers within the main chain. Only smaller units, such as bond lengths, bond angles, side groups, and small linkages, are capable of movement, while the motion of the chain segments remains inert, resulting in a glassy state of the polymer. With an increase in temperature, the energy available for thermal motion also rises. When the temperature reaches a threshold where this energy can overcome the rotational barriers of the main chain, the chain segments begin to move, and the polymer exhibits a highly elastic state. From the perspective of mechanical internal friction, when the motion of the chain segments is frozen, there is no mutual friction due to the absence of relative slippage between them, resulting in low internal consumption. At sufficiently high temperatures, the chain segments move freely and the interaction forces between them are diminished, indicating a minimal frictional force to be overcome during their sliding movement, and thus, internal consumption remains weak. It is during the transition from “thawing” to “free” movement that the chain segments exhibit a degree of mobility, but this is also when they must overcome high frictional forces, leading to increased internal consumption, which peaks at the glass transition temperature. At lower temperatures, the molecular thermal motion energy is insufficient for the chain segments to overcome the main chain's rotational potential, keeping them in a “frozen” state. It is posited that the relaxation process reflected in the loss factor is associated not only with the motion of the matrix's chain segments but also with the interfacial interactions between NQ solid particles or between the particles and matrix molecules. Furthermore, enhanced interfacial interaction between the matrix and NQ facilitates load transfer, reflecting a more finely integrated microstructure within the SLTP samples.^32,33^

The reinforcement of the polymer matrix by needle-shaped NQ is heavily reliant on the efficiency of interfacial load transfer. Typically, the intensity of the loss factor, represented by tan *θ* peak, serves as an indicator of the load transfer efficiency at the interface between the matrix and NQ. As illustrated in Fig. 6, the intensity of the tan *θ* peak escalates with an increase in driving frequency, which suggests that high-frequency vibrations enhance the load transfer efficiency at the interface. Concurrently, as the driving frequency increases, the tan *θ* peak of SLTP samples exhibits a shift towards higher temperatures, indicating that the SLTP sample manifests a high-frequency hardened viscoelastic behavior. The glass transition temperatures of both STP and SLTP samples have been ascertained through DMA analysis. The study elucidates a strong correspondence between the TMA inflection temperature and the DMA loss modulus softening temperature at lower frequencies. However, this relationship diminishes at higher frequencies. DMA tests also reveal that the storage modulus of the composites rises markedly, while their glass transition temperature (*T*_g_) decreases.

### 3.4 Dynamic structural damage

#### 3.4.1 Extreme crash resistance strength properties

**Fig. 7:**
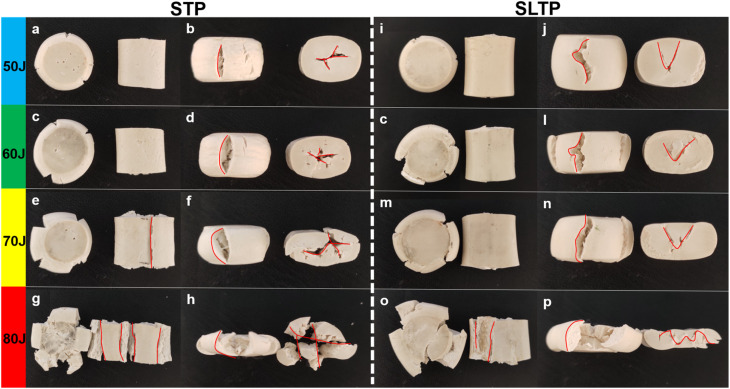
The axial and radial pictures after dynamic impact compression of STP sample and SLTP sample cylindrical particles.

To thoroughly examine the crack evolution and damage mechanisms of tensile damage in complex energetic material systems under varying conditions of collisional crush loading energy, and to offer valuable insights for the development of propellants with robust anti-explosive and anti-shock properties, this study utilizes falling hammers to characterize the mechanical structural properties of cylindrical energetic composite material particles. The propellant columns are subjected to a certain threshold of dynamic impact compression energy to simulate shock, with the aim of observing their response to external impact stimuli. The deformation and cracking state of the columns are analyzed to evaluate the damage behavior of the propellant columns under external impact stimulation. Fig. 7, captured by camera recording, displays the axial and radial views post dynamic impact compression of cylindrical particles from both STP and SLTP samples, facilitating further determination of the theoretical foundation for dynamic mechanical failure. Post-impact, the cylindrical particles exhibit a flattened shape in the stress direction, and instances of particle fracture are evident. Notably, as the axial and radial dynamic loading energy applied to the STP and SLTP samples is increased from 50 J to 80 J, the extent of tearing and fragmentation escalates markedly. Moreover, variances in the distribution orientation of the rigid NQ particles contribute to the number and direction of the resultant tears and fractures exhibiting significant differences, as evidenced in Fig. 7.

After subjecting cylindrical particles to dynamic impact compression energy, the emergence of cracks was noted in each trial, yielding distinct and significant outcomes. Dynamic impact compression tests conducted on regular cylindrical particles of both STP and SLTP samples revealed that the SLTP samples possessed a notably higher resistance to rupture under stress in both axial and radial directions compared to the STP samples, whether the impact was parallel or perpendicular to the molding axis. Furthermore, the test results indicate that the dynamic impact compression strength of the STP sample in the axial direction surpasses its strength in the radial direction substantially.

The observed crack morphology in the failed cylindrical particles from STP and SLTP samples is in stark alignment with the presumed rigid NQ arrangement morphology derived from previous assessments. In STP samples, the rigid NQ is uniformly oriented along the molding axis, whereas, in SLTP samples, the distribution of rigid NQ is random and lacks a defined orientation. These distinctions in NQ distribution are reflective in the fracture behaviors of the samples. For STP samples, the results corroborate that cracks swiftly propagate through numerous small voids (internal voids) located at the fragile interface between the rigid NQ and the composite matrix. These internal voids hinder the effective transfer of load from the matrix to the rigid NQ, acting as conduits for crack propagation and consequently leading to the tensile fracture of cylindrical particles. Conversely, the robust interface between the rigid NQ and the composite matrix in the SLTP samples enhances internal load transfer, prolongs the crack propagation route, and thereby serves as an effective deterrent to the advancement of cracks.

Upon examination of failed STP sample cylindrical particles, “L-shaped” cracks were identified on the radial outer surfaces, originating from axial dynamic impacts and extending through the upper and lower surfaces. Radial impacts resulted in “X-shaped” cracks within the axial end face plane, penetrating to the core. For the SLTP samples, similar axial impacts produced “Z-shaped” cracks across the radial outer surfaces, and radial impacts caused “V-shaped” cracks in the axial end plane area, also extending inward. The dynamic impact compression tests on both STP and SLTP samples revealed distinct damage mechanisms during loading. In the STP samples, the orientation of NQ parallel to the axial direction impeded the containment of radial crack expansion and collapse. The “X-shaped” penetration cracks observed on the end faces of STP samples are believed to arise from the convergence of random tensile cracks with lateral collapse slip zones, leading to the formation of “X-shaped” penetrative cracks. In contrast, the SLTP samples with randomly oriented NQ distribution exhibit a different pattern, where this random orientation may change crack trajectories and prevent the slip fractures caused by lateral collapses. The oriented arrangement of NQ within STP samples may alter the direction of crack propagation and mitigate slip fractures that typically occur due to lateral collapses. This effect is exemplified by the “V-shaped” penetration cracks on the axial end faces of the SLTP samples. When tensile cracks and collapse zones coalesce, the result is the fragmentation of cylindrical particles into numerous smaller pieces. Notably, further analysis reveals that STP sample particles tend to break down into smaller fragments than those from SLTP samples. Almost all of the smaller fragments from the failed STP samples align with the direction of NQ orientation, indicating that this fracturing pattern is influenced by the reorientation of NQ due to the molding process. For instance, with a dynamic impact compression energy of 80 J, STP sample particles post-radial impact are segmented into five longitudinally elongated strips along the radial direction. Meanwhile, under identical conditions, SLTP sample particles exhibit no significant separation, underscoring the difference in failure behavior between the two sample types.

In additional tests, it was observed that the number of fractured fragments from STP sample cylindrical particles typically exceeded that from SLTP sample particles, particularly at elevated levels of dynamic impact compression energy. During crush resistance tests with a dynamic impact energy range of 50–80 J, none of the SLTP sample cylindrical particles shattered completely, although they did flatten to various extents, exhibiting tensile rupture cracks at the end faces and around the periphery of the particles. The explanation for this phenomenon lies in the enhanced adhesion between the rigid NQ crystal clusters and the matrix interface. In the propellant formulation, the solid filler NQ contributes to the mechanical fortification of the system. A high-quality interfacial bonding effect may produce more physical cross-linking sites between the NQ and the binder when the proportion of the filler NQ particle system remains constant. This heightened resistance to chain segment movement within the composite system allows for the effective dispersion and transfer of impact stresses, mitigating damage to the composite structure, and consequently, significantly bolstering crush resistance.

Elastic mechanical analysis identifies radial tensile fracture, or axial splitting, as a frequent form of damage resulting from collisional crush loading, primarily due to the Poisson effect. When subjected to rapid axial compression, a sample experiences uniform internal tensile stress in all directions perpendicular to the load. This stress causes the sample to lengthen and stretch diametrically, producing tangential tensile stress. If this stress exceeds the tensile strength of the sample particles, it leads to stretch cracks and subsequent collapse, causing the sample to break into smaller fragments.

**Fig. 8:**
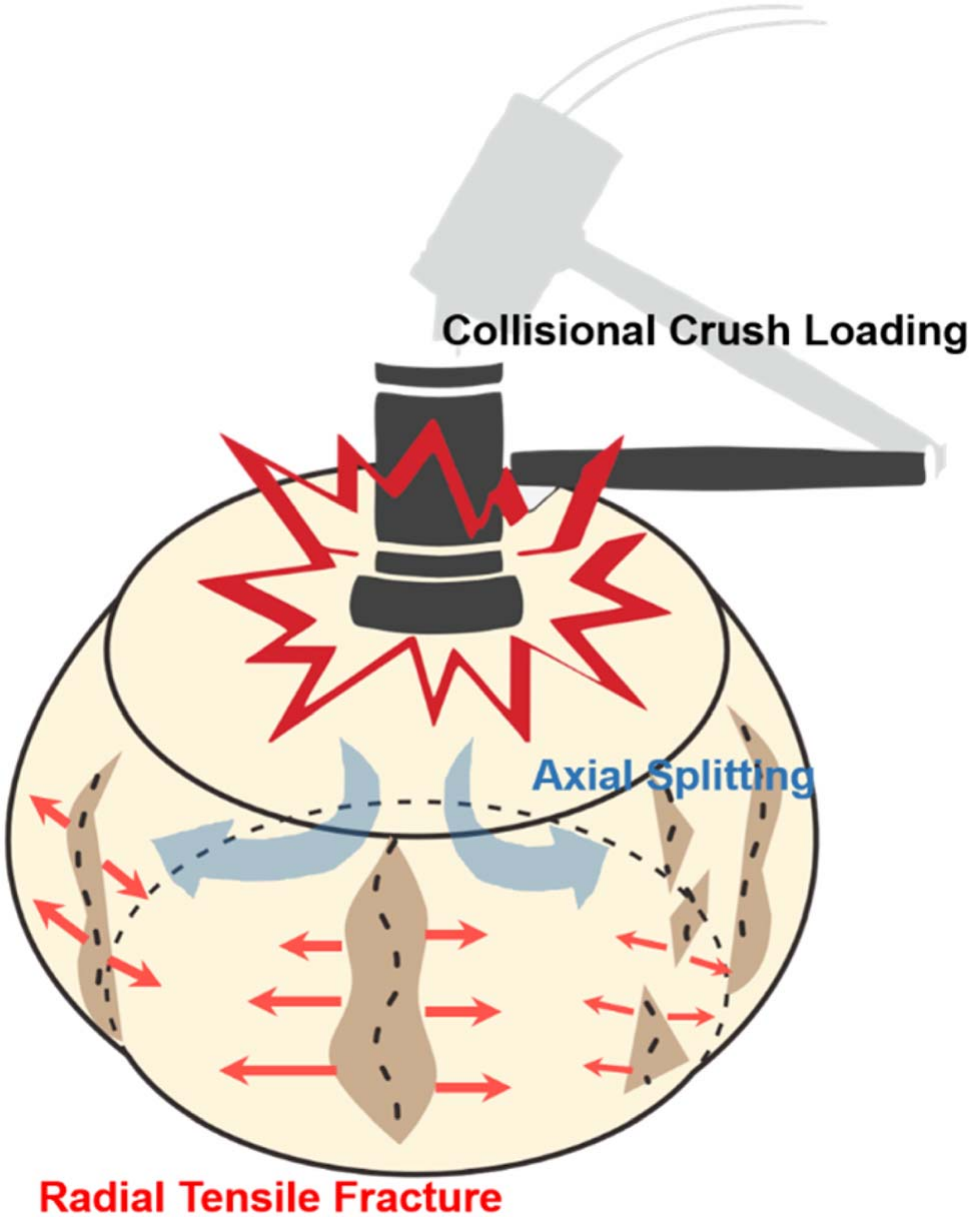
Directional effects and interrelationships of collisional crush loading, radial tensile fracture, and axial splitting.

According to elastic mechanics analysis, the more common mode of damage under collisional crush loading is radial tensile fracture (or axial splitting) caused by the Poisson's effect, as shown in the illustration. When the sample is rapidly compressed along the axial direction (as indicated by the black falling hammer impact), the compressive load generates uniform internal tensile stress on all surfaces perpendicular to the loading direction (shown by blue arrows). The sample undergoes elongation in the diameter direction, and the compressive load creates tangential tensile stress. Due to the tensile stress exceeding the tensile strength of the sample particles, there will be the occurrence of tensile cracks and collapse around the sample particles, resulting in the fragmentation of the sample into smaller particles (indicated by red arrows) (Fig. 8).

Notably, such a standard damage pattern is exhibited solely by STP samples, which suggests a discrepancy in bond strength within the sample's internal structure; the axial bonds are stronger than the radial ones. Moreover, a distinct pattern of damage was observed in this experiment, as exemplified in Fig. 7. The oblique cracks on the sample surface depicted in Fig. 7 signal that the internal failure, governed by maximum tensile strain, has not advanced to the critical state of total fragmentation, hence the overall structure of the sample remains largely intact.

In this instance, an alternative mechanism governs the disintegration of SLTP sample particles. Examination of the test data revealed that despite the considerable variation in collisional crushing energy, all fracture surfaces were oriented at an angle relative to the axial line of the specimen particle, indicating a clear non-parallelism. These deviation angles likely signify the intrinsic attributes of the SLTP samples' internal structure, which possesses greater resilience against radial extension due to the Poisson effect. Consequently, stress transmission within SLTP samples transitions from a unidimensional to a tridimensional form. The radially constrained bidimensional stresses in the SLTP samples, resulting from vertical impact crushing loads, cause angular deflection on the circumferential fracture surfaces. This angular deflection facilitates the dissipation and release of energy, such as the dispersion of circumferential fracture stress waves at the boundaries of the cracks.

As delineated, the fundamental mechanism underlying the mechanical structural failure of rigid NQ particle and polymer matrix composites is stress-induced phase separation. These rigid NQ particles facilitate the partial absorption and dissipation of microstrain within the polymer chain network. From a microscopic perspective, the dissipation of mechanical energy within the rigid NQ particle–polymer matrix composite is predominantly governed by the three-dimensional cross-linking of this network, which plays a more crucial role than mere one-dimensional axial cross-linking. Given that rigid NQ particles do not exist as free small molecules but rather as physically interconnected, elongated, needle-shaped 3D networks, the reconfigured rigid NQ particle packing network becomes an essential conduit for stress mitigation and conveyance among the rigid NQ particles themselves, as well as between these particles and the polymer composite matrix. Amidst microstrain, the physical bonds within these 3D interwoven crosslinks not only mitigate mechanical energy but also preserve the cohesiveness of the 3D interlaced network for both rigid NQ particles and the polymer chain network, ensuring efficient stress transference between them. This process precludes the compromise of structural stability in the energetic composite materials prior to any macroscopic fracturing. In summary, the SLTP samples demonstrate a heightened macroscopic resilience to continuity cleavage, buttressing structural integrity and continuity to accommodate the intricate load transfer trajectory.

### 3.5 Correlation between particle orientation and structural strength

The CT scan visualization elucidates the intricate orientation state of the NQ crystals. Leveraging CT data for assessment offers the significant benefit of enabling the direct derivation of even fourth-order orientation tensors from the imaging data.^34,36^ Consequently, to validate the strong correlation between the spatial modulus of triple-base propellants and NQ crystal orientation, this study employs high-resolution CT imaging to ascertain the three-dimensional configuration of the NQ crystal-augmented triple-base propellants and to parameterize the spatial configuration of each NQ crystal. We introduce a structural tensor-based computational method that hinges on an iterative process of splitting and merging individual NQ crystals to ascertain NQ characteristics: orientation, position, diameter, and length. We postulate that a singular NQ crystal can be modeled as a cylinder with a length *L*, diameter *Φ*_D_, and orientation *P*. Here, *α* denotes the angle between the NQ needle and the *Z* direction (indicative of extrusion molding direction), while *β* represents the angle between the projection of the NQ needle in the *X*–*Y* plane and the *X*-axis. The effective stiffness tensor *C* for a particulate composite is typically expressed by the ensuing eqn (1).1
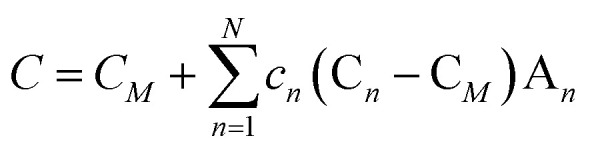
where *c* denotes the volume fraction of the needle NQ; *A* denotes the fourth-order strain localization tensor; *N* corresponds to the number of needle NQ within the volume element under consideration; and *M* denotes the matrix phase.

It is assumed that each NQ crystal is embedded in an infinite homogeneous medium with the same properties as the base material P*, leading to the following expression for the strain localization tensor *A*_*n*_ (eqn (2)):

2 *A*_*n*_ = *A*(C*,*C*_*n*_, *Z*_*n*_) = (*I* + P*(*C*_*n*_ − C*))^−1^

In this representation, the strain localization tensor *A*_*n*_ depends on the substrate material P*, the NQ crystal stiffness *C*, and the spherical approximation parameter *Z* of the NQ crystal, which includes the orientation of the NQ crystal axis.

The outcomes of the structural tensor analyses suggest that the elastic modulus of the energetic composite materials incrementally rises as the angle between the externally applied force and the rigid NQ crystal clusters diminishes. This observation is attributable to the composition of the energetic materials, which consist of block copolymers with soft nitrocellulose chains and rigid NQ crystal cluster chains. The supple nitrocellulose chains, which exhibit considerable plastic deformation, are capable of dispersing the force between the nitroguanidine crystal cluster material and the molecular chains of nitrocellulose. Consequently, the angle between the additional force and the rigid NQ crystal cluster is instrumental in significantly mitigating the shear stress imposed on the NQ crystal clusters, thereby ensuring that the shear stress is more evenly distributed.

**Fig. 9:**
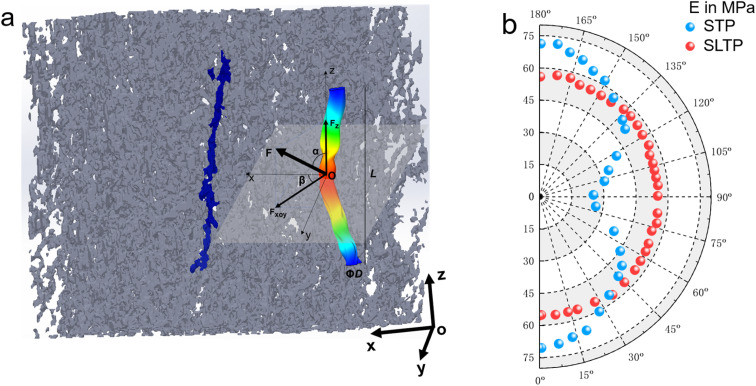
Theoretical elastic modulus *versus* needle NQ spatial angle for the triple base propellants enhanced by needle-shaped NQ structure.

Fig. 9 indicates that the elastic modulus of the SLTP samples remains comparatively consistent across all measured angles, exhibiting stability and suggesting isotropic structural properties. Specifically, the modulus for SLTP samples is reported to be within 53.8–57.3 MPa for angles ranging from 0° to 180°, leading to the inference that the modulus is uniform in all directions. In contrast, STP samples displayed a variation in elastic modulus with orientation: presenting the lowest modulus value in the horizontal direction and the highest in the vertical. The modulus of STP samples decreased with an increase in angle from 0° to 90° and increased as the angle grew from 90° to 180°.

The observed maximum elastic modulus for the samples is 78.4 MPa at both 0° and 180°, while the minimum modulus is recorded at 22.8 MPa at 90°. These values underscore the distinctly anisotropic structural properties of the material. Notably, the SLTP samples demonstrate pronounced central symmetry in their elastic modulus across the entire spectrum of angles. Conversely, the STP samples manifest regular axisymmetry throughout the full range of angles, with an axis of symmetry at 90°, indicating a marked angular dependence.

It is particularly noteworthy that the elastic moduli of the STP and SLTP samples converge at two angular points within the full range, specifically at 38° and 143°, where they both register a modulus value of 55 MPa. Additionally, within the angular span of 38° to 143°, the elastic modulus of the SLTP samples significantly exceeds that of the STP samples. Outside this range, namely between 0° to 38° and 143° to 180°, the elastic modulus of SLTP samples is marginally lower than that of the STP samples. In essence, this implies that the SLTP sample possesses a higher elastic modulus across a broader angular region when compared to the STP sample within the entire angular spectrum.

## 4 Conclusion

In the manufacture of propellants, we have refined a solvent-free process route that is both environmentally benign and highly productive. This route employs a high-temperature and high-pressure plasticization integration method to guarantee a homogeneous distribution of needle-shaped nitroguanidine crystals within the propellant and robust interfacial adhesion. Distinctive features of this methodology include minimal material usage, a shortened processing pathway, low emissions, and enhanced efficiency, stability, and reliability. The resulting products (SLTP) from this process exhibit higher relative density, precise dimensional accuracy, superior mechanical properties, and an expanded range of environmental suitability. The details are as follows.

Regarding macroscopic physicochemical characteristics, the SLTP samples demonstrated an increased extrusion swelling rate of approximately 2.75%, reduced volatile solvent residuals to around 0.03 wt%, diminished moisture residual concentration to about 0.49 wt%, and an elevated apparent density reaching approximately 1.68 g cm^−3^.

During the examination of microstructural morphology, it was observed that the SLTP samples exhibit a mutually supportive three-dimensional spatial structure. The NQ crystals within these samples are more densely and effectively organized at the bonding interface with the matrix.

The thermomechanical response of the materials is delineated through static thermomechanical analysis (TMA) and dynamic mechanical analysis (DMA). Static TMA revealed that the glass transition temperature of the SLTP samples remained stable irrespective of the plane of detection. The coefficients of linear expansion for the three axes displayed a high degree of overlap and remarkable consistency across the entire temperature spectrum. Dynamic mechanical property evaluation indicated that the SLTP samples maintained a more constant and lower glass transition temperature, which correlates well with their performance, thereby broadening their operational temperature range. Additionally, the energy storage modulus suggested superior microbinding within the SLTP samples' internal structure. Collectively, these thermomechanical responses indicate that the SLTP samples possess a highly uniform isotropic structure, with reduced propensity for brittleness and lower glass transition temperatures, enhancing their environmental adaptability.

Dynamic structural damage testing, exemplified by extreme crush resistance, reveals key performance indicators. Fragmentation resistance assessments have determined that the robust interfacial bond between the rigid NQ and the composite matrix in the SLTP samples bolsters load distribution within the material and extends the trajectory of crack propagation. This intervention effectively curtails the expansion of cracks, thereby facilitating the efficient dispersion and conveyance of impact stresses. As a result, there is a diminution of damage to the composite structure, which translates into a marked elevation in crush resistance. Summarily, dynamic structural damage tests underscore that the SLTP samples macroscopically present a high resistance to continuity splitting. They are adept at maintaining structural integrity and continuity, ensuring the integrity of complex load transfer pathways, and providing effective dynamic impact and fragmentation resistance.

Finally, a structural mechanics model for nitroguanidine micron rod reinforced triple-base propellant was constructed. This model quantitatively characterized the dependency of structural strength on the orientation of the solid-filled NQ crystals for both types of propellants, corroborating the viability and precision of the theoretical framework. Concurrently, it furnishes a theoretical foundation for understanding the unique internal organization and mechanical phenomena through the lens of theoretical computation.

## Author contributions

Yao Zhu: idea; methodology; writing – original draft preparation; revised manuscript. You Fu: conceptualization; reviewing; revised manuscript. Bin Xu: formal techniques to analyze or synthesize study data. Zhitao Liu: second corresponding author, supply for the experimental platform; writing – editing. Feiyun Chen: supply for the experimental platform, funding provider. Xiaoan Wei: supply for the experimental platform, reviewing and editing. Xin Liao: first corresponding author, conceptualization; raw materials provider; writing – reviewing and editing; funding provider; supervision (oversight and leadership responsibility for the research activity planning and execution, including mentorship external to the core team).

## Conflicts of interest

The authors have no competing financial interests or personal relationships that could have influenced the work. They have no professional or personal interest in any product, service, or company that could affect the position presented in the manuscript. All authors have approved the manuscript for publication.

## Supplementary Material

RA-013-D3RA04828D-s001
