# Supplementary material for: Advanced fabrication approach for innovative triple base propellants with enhanced continuous fracture resistance
Source: RSC Adv. 2023 Dec 6;13(50):35602–16. doi: 10.1039/d3ra04828d (PMC10698641; doi:10.1039/d3ra04828d)
Supplement: RA-013-D3RA04828D-s001 [file RA-013-D3RA04828D-s001.pdf]

Electronic Supplementary Material (ESI) for RSC Advances.
This journal is © The Royal Society of Chemistry 2023

Supporting Information

Advanced Fabrication Approach for Innovative Triple Base Propellants with Enhanced Continuous Fracture Resistance

Yao Zhu[ab#], You Fu[ab], Bin Xu[ab], Zhitao Liu[ab*], Feiyun Chen[ab], Xiaoan Wei[ab], Xin Liao[ab*]

a School of Chemistry and Chemical Engineering, Nanjing University of Science and Technology, Nanjing, Jiangsu 210094, China

b Key Laboratory of Special Energy Materials, Ministry of Education, Nanjing, Jiangsu 210094, China

First author: E-mail: howtosci@163.com (Yao Zhu)

* Corresponding E-mail: liaoxin331@163.com (Xin Liao); lzt03304645@163.com (Zhitao Liu)

1 Characterization

1.2 Scanning Electronic Microscopy

Scanning electron microscopy (SEM) images were obtained using Zeiss Sigma 300 (Carl Zeiss Ltd, Cambridge, UK) with a resolution of 1 nm at an accelerating voltage of 15 kV to characterize the cold brittle fracture surfaces morphology and microstructure of the tested propellant samples from different dimensions, and also examine the needle-shaped NQ crystal-matrix-bonding interface and the NQ crystal distribution in the specimens. The cold brittle fracture surface is achieved by directional splitting after rapid cooling of the grains in liquid nitrogen. Thus the ensuing cracks exposed a fresh surface, neither stretched nor compressed, and the internal structure of the material was revealed with little disturbance due to the brittleness of the low temperature fracture. All the samples were coated under vacuum with a thin gold film for a period of 120 seconds under a vacuum of 10^{-6} Pa.

1.3 Apparent Density Measurement

The apparent density of propellant particles was measured by the density bottle process, and the impregnating solution was the deionized water. The ambient temperature and humidity levels were kept at constant values of 25°C and 76%, respectively, during the test. In order to prevent the effects of prolonged crystallization followed by aging deterioration processes occurring in the high-energy polymer matrix phase, all samples were measured over a period of two days. The apparent density can be calculated by **Equation S1.**

$$\rho = \frac{m\rho_t}{m_1 - m_2 + m} \quad \text{(S1)}$$

where ρ is the apparent density of the propellant particles at 25°C in g/cm^3; m is the mass of the propellant particles in g; ρ_t is the density of deionized water at 25°C in g/cm^3; m_1 is the mass of the density bottle filled with deionized water in g; and m_2 is the mass of the density bottle filled with impregnating liquid and propellant samples in g.

For each sample, air bubbles bound to the material surface are removed using negative pressure of 0.1 MPa and high frequency mechanical vibration, and the

process is continued for at least 30 min to ensure the stability of the results. The process was repeated 3 times for removing air bubbles. Five parallel results were measured for each sample. The average value to be used as the result.

1.4 Static mechanical thermal analysis (TMA)

The theory of TMA measurement is predicated on the distortion of the material, which will be acquired as a result of the change in temperature [16]. The linear coefficient of thermal expansion of a material can be measured by determining the deformation of the material over a certain temperature range. **Fig. S1** shows the principle diagram of the TMA. The sample was fabricated by smoothed parts selected from a solid cylindrical gun propellant, the samples were cubic pillars with a flat surface and the length, width and height were 10 ± 0.1mm, respectively. The static thermo mechanical analysis of the sample was measured by TMA/SDTA 841e (Mettler Toledo, Switzerland) within the range of 224-354 K at a heating rate of 5 K/min in a dry nitrogen atmosphere[17][18][19]. The length resolution and contact force range are 0.5nm and 0.1N-1N, respectively. The coefficient of thermal expansion can be formulated as **Equation S2.**

$$\alpha_L = \frac{dL}{dT} \cdot \frac{1}{L} \qquad \text{(S2)}$$

where α_L, dL, L, dT are the linear coefficient of thermal expansion, the length change, the original length and the corresponding temperature change of the material, respectively.

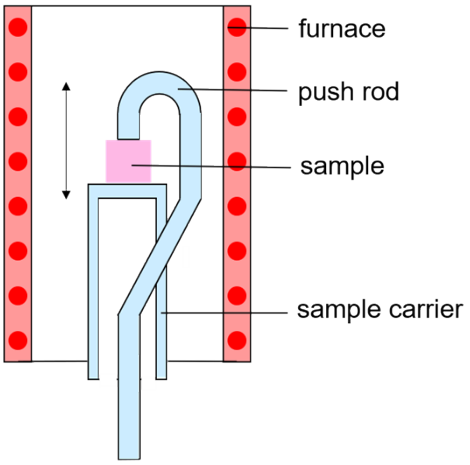

Fig. S1 Schematic diagram of static mechanical thermal analysis (TMA) test

1.5 Dynamic Mechanical Thermal Analysis (DMA)

In this work, the dynamic mechanical thermal behavior of the cylinders with the large-size triple-base propellants was observed by Gabo Eplexor® DMA (Gabo Qualimeter, NETZSCH, Shanghai, China) equipped with a 500 N load cell in multi-frequency-strain mode. The test was conducted under displacement control using dynamic load structure with a three-point bending configuration and the distance between the supports was 40 mm. Based on the temperature ramp sweep testing method. The applied temperature range was between -55 and 85℃ at a heating rate of 2.0 K/min under nitrogen atmosphere. The isothermal dwelling time at -55 ℃ was 30 min in order to stabilize the microstructure of the material. The applied oscillation strain was 0.05% with 10 mN contact force at a dynamic amplitude of 20 μm. In such experiments performed under sine sinusoidal stress with scanning frequency range of 10hz, 20hz and 30hz respectively. Three parallel samples were performed for each formulation. As part of the measurements, the storage modulus (E′), loss modulus (E″) and loss factor (tanδ) were mapped out and then compared over the range of temperatures investigated. Tanδ is an indicator of the energy consumed by the viscous mechanism relative to the energy stored in the elastic component.

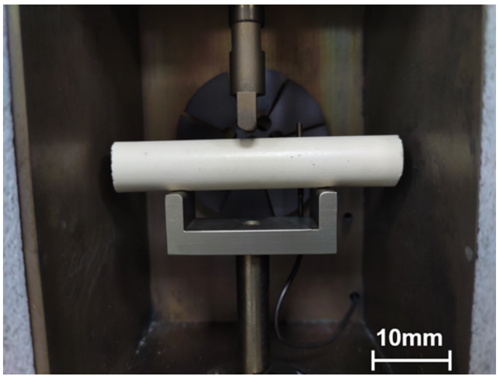

Fig. S2 Photograph of Dynamic Mechanical Thermal Analysis (DMA) test system

1.6 Collision Crushing Strength Tests

The mechanical collision crushing strength properties of propellant grains under the temperature of 25℃ were evaluated using a standard Bundesanstalt für Materialprüfung (BAM) Fall-hammer device (OZM, Czech Republic, model No. BFH-10) with a drop-weight of 5 kg, a collection device and a protection test chamber. The sample to be tested was a cylindrical pillar of 10 mm in diameter with

parallel top and bottom surfaces, and all pillars were held at 25°C for 4 hours before the impact test. The impact direction of the test is parallel to the axial and radial directions of the sample, respectively. The impact kinetic energy of the falling hammer is 50J, 60J, 70J and 80J. The broken flakes are collected after each impact with a collector with a protective barrier. The record was repeated five times for each test sample to ensure the accuracy of the test results. Generally, the reproducibility of these examinations is within 90%. The samples whose fracture surfaces deviated significantly from the orthogonal relationship with the notched surface of the samples were not considered in the final analysis.
